# A Case Report on Early-Onset Misophonia in a Bangladeshi Pediatric Patient

**DOI:** 10.7759/cureus.86245

**Published:** 2025-06-17

**Authors:** Humayra S Hridi

**Affiliations:** 1 Department of Child and Adolescent Psychiatry, LifeSpring Consultancy Limited, Dhaka, BGD

**Keywords:** bangladesh, misophonia, ocd, pediatric psychiatric disorder, selective sound sensitivity syndrome

## Abstract

This case report describes a nine-year-old Bangladeshi boy with misophonia symptoms beginning at three years old, an underreported condition in children. He exhibited strong emotional reactions to specific sounds made by his mother, such as yawning and breathing, which later extended to visual triggers, including her nose-scratching. He attempted to stop these triggers through physical actions. Autism was ruled out. A brief trial of sertraline, risperidone, and behavioral therapy showed minimal benefit and was discontinued. In the fifth year, compulsive hand-washing emerged. Family history revealed parental OCD. On reevaluation, he was diagnosed with misophonia and emerging OCD. Treatment included selective serotonin reuptake inhibitors, cognitive behavioral therapy with exposure and response prevention, relaxation techniques, and follow-up. This case underscores the potential for early-onset misophonia with auditory and visual triggers, its overlap with OCD, and the need for early diagnosis, attention to comorbidities, and individualized, ongoing care.

## Introduction

Misophonia is a condition gaining increasing recognition globally, affecting both adults and children. A systematic review has reported that its prevalence among children and adults ranges from 5% to 34.67% [1]. Misophonia is a strong aversive reaction to specific trigger stimuli, commonly ordinary sounds such as chewing, yawning, breathing, or sighing, as well as repetitive movements like leg tapping, scratching, or typing [2,3]. It is distinct from phonophobia, the irrational fear of sound, and hyperacusis, an intolerance to sound volume [4,5]. A systematic study concluded that the diagnostic model proposed by Dozier et al. [6], which builds upon the earlier work of Schröder et al. [4], provides a promising framework for understanding misophonia. This model encompasses key features that identify misophonia, including conditioned sensory triggers, immediate physical and emotional responses, recognition of the disproportionate nature of these reactions, and avoidance behaviors that significantly disrupt daily functioning. The specific misophonic triggers can vary widely between individuals, suggesting that these responses are shaped by personal experiences and contextual factors [3]. Recent findings indicate that misophonia can begin as early as three years of age, although it more commonly emerges in adolescence or adulthood [5]. In pediatric populations, it is often misinterpreted as other psychiatric disorders [7,8]. According to a study by Guzick et al. [8], approximately 80% of children with misophonia have at least one co-occurring psychiatric condition. The most frequent comorbidities include anxiety and depressive disorders, particularly obsessive-compulsive disorder (OCD), which may indicate a more severe and treatment-resistant presentation [5,8]. Despite increasing global awareness, misophonia remains underdiagnosed in children, especially in low- and middle-income regions such as South Asia. There is a critical need for case reports from these regions to raise clinical awareness, support early diagnosis, and guide culturally informed management strategies. This report presents a rare pediatric case of misophonia in Bangladesh, characterized by early onset, multiple sensory triggers, obsessive behaviors, and comorbidity with OCD.

## Case presentation

A nine-year-old Bangladeshi boy, the only child in his family, reported experiencing marked affective disturbances and inappropriate behavioral reactions over the past six years in response to certain sounds made by his mother. These included burping, sneezing, yawning, breathing, and even speaking from the side or behind him, with the specific triggers occurring at different points in time. The symptoms started at three years old and got worse over time. By the time he was six years old, visual cues, such as his mother scratching her nose, also started to cause him distress. There was no history of tinnitus. Initially, the child exhibited emotional outbursts and tantrums soon after hearing the sounds or made attempts to leave the room. Over time, however, these behaviors evolved into compulsive actions. These included physically pulling his mother’s nose after she scratched it or attempting to touch her cheek with his ear after she made certain sounds. He explained that the action helped him overcome the burning sensation he experienced from those sounds. At first, his mother was the only trigger for him; later on, he reacted similarly to his father, who occasionally came to stay with him from abroad. He did not respond to others or educational settings in the same way as his peers, as his peers never triggered a reaction in him. His academic performance was good. He showed modest social timidity, but did not have any impairment in social communication. His speech and cognitive milestones were normal. When he was five years old, he first visited a psychiatrist and was prescribed risperidone (1 mg) due to frequent temper tantrums and anger outbursts in response to sound triggers, but the medication was later discontinued. When the symptoms increased, sertraline was subsequently introduced at seven years old and titrated up to 50 mg. He also received two to three sessions of behavioral therapy, which led to slight but insufficient improvement, and the treatment was subsequently discontinued by the parents. After two years, the boy started washing his hands obsessively (20-30 times a day), which he was unable to explain. Family history was notable for both parents' history of OCD.

On mental status examination at the time of presentation, he appeared visibly anxious and agitated. His intelligence was average. He had obsessive thoughts of dirt and contamination. There was no evidence of suicidal ideation or intent. He demonstrated partial insight into his condition. He was diagnosed with misophonia as per the diagnostic model proposed by Dozier et al. [6] and emerging OCD. A revised management plan included fluoxetine, propranolol, and cognitive behavioral therapy (CBT) with exposure and response prevention (ERP), along with progressive muscular relaxation therapy (PMRT). Some advice on using noise-cancelling headphones or earplugs was given as a coping strategy. The child was advised to stay with his parents, and the parents were psychoeducated on understanding misophonia and sensory processing, identifying and managing triggers, implementing coping strategies like advice on healthy adaptive accommodation, creating a supportive environment, and promoting emotional regulation. Ongoing follow-up was recommended to monitor treatment response and to assess for the emergence of additional internalizing or externalizing symptoms.

## Discussion

This case provides valuable clinical insights into one possible manifestation of misophonia in children and highlights potential treatment approaches based on existing evidence. However, treatment response is not the primary focus of this report and has therefore not been discussed in detail.

First, this case demonstrates symptom onset as early as three years old, whereas most reported cases of misophonia begin after age seven. This supports recent pediatric findings suggesting that early childhood misophonia may be more common than previously thought, though often misinterpreted as behavioral problems or oppositionality. Additionally, the same study indicates that misophonia in children may predict future internalizing and externalizing disorders, as well as academic challenges [5].

Second, despite the fact that misophonia is typically linked to particular auditory stimuli, the prevalence of visual triggers, like nose-scratching in this instance and rubbing his ear with his mother’s cheek, after she made certain sounds, emphasizes the phenomenon of misokinesia, which is characterized by aversive reactions to specific visual cues. This condition often coexists with misophonia and emphasizes the necessity of a more comprehensive conceptualization of triggering stimuli [2,6].

Third, misophonia shares overlapping symptoms with several psychiatric and neurodevelopmental conditions, such as OCD, specific phobias, phonophobia, autism spectrum disorder, and hyperacusis, often leading to diagnostic confusion and underdiagnosis. It is also commonly comorbid with psychiatric disorders like depression, OCD, anxiety, autism, and attention-deficit/hyperactivity disorder, as well as neurological conditions such as Tourette’s syndrome and auditory system disorders [2,3,5].

Approximately 13% of children diagnosed with misophonia also meet the diagnostic criteria for OCD [5]. Although misophonia and OCD share underlying neurobiological mechanisms, particularly involving emotional regulation and limbic system dysregulation, misophonia is characterized by distinct sensory-triggered aversive responses (Table 1) [9], highlighting the necessity for precise differential diagnosis and individualized treatment approaches [8]. In the present case, treatment initially targeted OCD exclusively, which was regarded as the primary diagnosis, while misophonia remained unrecognized; this oversight likely contributed to suboptimal therapeutic response and clinical deterioration. The literature underscores a significant association between misophonia and OCD, reinforcing the importance of addressing both conditions in diagnostic and therapeutic planning [7,10]. Additionally, a positive familial history of OCD in both parents suggests a possible genetic or hereditary component in misophonia, which may inform management strategies by prompting early screening for OCD and related symptoms in affected patients [3].

**Table 1:** Clinical characteristics distinguishing misophonia and OCD based on information from various studies OCD: obsessive-compulsive disorder; DSM-5: Diagnostic and Statistical Manual of Mental Disorders, Fifth Edition Source: [4-6,9-11]

Feature	Misophonia	OCD
Definition	A condition where there is a strong emotional reaction (e.g., anger and disgust) to specific sounds	A disorder involving unwanted, intrusive thoughts (obsessions) and repetitive behaviors (compulsions)
Disorder/syndrome	Variable opinions as per different studies	Disorder
Diagnosis category	Not officially recognized in DSM-5 as a distinct disorder	Classified as a recognized mental disorder in DSM-5
Main focus	External sensory stimuli (sound-specific)	Internal thoughts and urges (mental patterns)
Trigger	Specific sounds, visual triggers like kinesthetic movements (e.g., chewing, tapping, and breathing)	Obsessional thoughts (e.g., fear of germs and need for symmetry), urges, and images trigger compulsive actions
Emotional response	Immediate irritation and anger	Anxiety or distress from intrusive thoughts
Behavioral response	Avoidance or escape from triggering sounds; sometimes verbal/physical outbursts	Compulsions are performed to reduce anxiety (e.g., checking, counting, and washing).

Although research on treatment options for managing OCD in children is limited, existing evidence indicates that addressing misophonia within the context of OCD requires a multifaceted treatment approach. This involves a combination of pharmacological and psychological interventions [2,7,12]. Selective serotonin reuptake inhibitors, such as fluoxetine [12], have shown effectiveness, especially when paired with evidence-based therapies like CBT and ERP [7,11]. Supportive strategies, such as mindfulness, relaxation techniques like PMRT [13], and practical tools like noise-canceling headphones, can further support symptom management [7]. This comprehensive treatment plan was implemented in the current case. In this case, the family was advised to adopt accommodation strategies in a healthy and adaptive manner, ensuring that avoidance behaviors were not reinforced, as supported by existing research [14].

Finally, the chronic and escalating nature of the child’s symptoms, along with associated emotional and behavioral disruptions, highlight the importance of early diagnosis along with maintaining a longitudinal follow-up [5,7]. In young patients exhibiting atypical sensory responses, this case highlights the necessity for heightened clinical awareness, comprehensive evaluation across sensory modalities, and individualized treatment planning. This underscores the necessity for a comprehensive assessment to elucidate the specific sensory modalities involved and to identify the contextual factors that modulate the patient's responses. Such an approach is essential for developing targeted interventions and enhancing our understanding of the underlying mechanisms contributing to atypical sensory processing, while also emphasizing the need for developing and validating culturally appropriate assessment tools to improve diagnosis and treatment monitoring in this population.

## Conclusions

This article presents a rare case of early childhood-onset misophonia in a Bangladeshi child, characterized by both auditory and visual triggers and complicated by co-occurring OCD. Recognizing misophonia is crucial across multiple disciplines-including pediatrics, audiology, and psychiatry-to foster a comprehensive understanding and collaborative approach to diagnosis and management. The case underscores the clinical challenge of differentiating compulsive behaviors arising from misophonia versus those associated with primary OCD. It highlights the need for early recognition, comprehensive symptom assessment-including multimodal sensory triggers-and regular longitudinal psychiatric follow-up to mitigate long-term psychological impacts. Clinically, this case also emphasizes the importance of screening for comorbid OCD in pediatric misophonia presentations. Future research should investigate the effectiveness of various treatment approaches in broader patient populations with misophonia and comorbid OCD to support evidence-based care.
